# Relationships among Facial Mimicry, Emotional Experience, and Emotion Recognition

**DOI:** 10.1371/journal.pone.0057889

**Published:** 2013-03-25

**Authors:** Wataru Sato, Tomomi Fujimura, Takanori Kochiyama, Naoto Suzuki

**Affiliations:** 1 The Hakubi Project, Primate Research Institute, Kyoto University, Aichi, Japan; 2 ERATO, Okanoya Emotional Information Project, Japan Science and Technology Agency, Wako, Saitama, Japan; 3 Department of Psychology, Graduate School of Letters, Dosisha University, Imadegawa, Kamigyo-ku, Kyoto, Japan; Aalto University, School of Science, Finland

## Abstract

**Background:**

The relationships between facial mimicry and subsequent psychological processes remain unclear. We hypothesized that the congruent facial muscle activity would elicit emotional experiences and that the experienced emotion would induce emotion recognition.

**Methodology/Principal Findings:**

To test this hypothesis, we re-analyzed data collected in two previous studies. We recorded facial electromyography (EMG) from the corrugator supercilii and zygomatic major and obtained ratings on scales of valence and arousal for experienced emotions (Study 1) and for experienced and recognized emotions (Study 2) while participants viewed dynamic and static facial expressions of negative and positive emotions. Path analyses showed that the facial EMG activity consistently predicted the valence ratings for the emotions experienced in response to dynamic facial expressions. The experienced valence ratings in turn predicted the recognized valence ratings in Study 2.

**Conclusion:**

These results suggest that facial mimicry influences the sharing and recognition of emotional valence in response to others' dynamic facial expressions.

## Introduction

Facial mimicry refers to the automatic production of a facial expression that is congruent with an observed facial expression [1]. Previous psychophysiological studies using facial electromyography (EMG) have empirically supported the existence of facial mimicry. For example, Dimberg [2] showed that mere photographic presentations of angry and happy facial expressions induced corrugator supercilii muscle activity (brow lowering actions, prototypical in angry facial expressions) and zygomatic major muscle activity (lip-corner pulling actions, prototypical in happy facial expressions), respectively.

The psychological processes related to facial mimicry have long been of great interest to scholars. Classically, Nietzsche [3] proposed that the observation of others' emotional facial expressions automatically induced facial mimicry, which led to an emotional experience that then produced the recognition of the emotion experienced by other people. Several classical (e.g., [4]) and contemporary (e.g., [5], [6]) psychological researchers have also proposed similar models with refined details.

Several previous experimental studies have provided supportive evidence for parts of Nietzsche's [3] model. For example, Bush et al. [7] showed that intentional control of facial muscle activity modulated subjective emotional reactions while participants viewed comedy films. Niedenthal et al. [8] reported that intentional control of facial muscle activity affected judgments about facial expressions used as stimuli. Richards et al. [9] found that the manipulation of emotional mood influenced the recognition of the emotion portrayed in stimulus facial expressions. These data suggest the existences of links between facial mimicry, the elicitation of emotion, and the recognition of emotion.

However, no evidence supporting the full model has been provided. Only a few studies have tested the relationships among facial mimicry, emotional experience, and emotion recognition, and all these studies reported null findings [10]–[12]. Whereas Gump and Kulik [11] used raters' judgments of videotapes, Blairy et al. [10] and Hess and Blairy [12] used facial EMG recordings, which consitute a more sensitive measure of viewers' reactions. These researchers recorded the activity of several facial muscles, including the corrugator supercilii, while participants observed photos or videos of facial expressions depicting several negative and positive emotions. The participants also used a scale to measure the intensity of several emotions they both experienced and recognized in response to stimulus facial expressions. The researchers calculated the accuracy of emotion recognition in a binary manner (correct or incorrect) using the highest-rated categories and calculated correlations or regressions (cf. [13]) between the variables. The results did not provide clear support for the relationships between facial mimicry and the emotion elicited, facial mimicry and the emotion recognized, or the emotion elicited and the emotion recognized. Thus, the full model proposed by Nietzsche [3] has remained empirically unproven.

This issue can be clarified by studies investigating relationships between facial EMG activity and emotional experience that arise in response to emotional scenes [14]–[18]. These studies assessed emotional experience based on the dimensional view that subjective emotion can be depicted using two dimensions: valence, which represents a qualitative component ranging from negative to positive, and arousal, which reflects the intensity of emotions ranging from low to high (cf. [19]). These studies have consistently reported that facial EMG activity was related to ratings of experienced valence. Specifically, the corrugator supercilii and zygomatic major muscles showed negative and positive relationships with valence ratings from negative to positive, respectively. Based on these data, we hypothesized that facial EMG activity in response to emotional facial expressions would be related to the experienced and recognized emotions represented by valence ratings.

The use of a dynamic or static presentation condition may be relevant in the investigation of this issue. Dynamic facial expressions are more natural and powerful than are static ones. Consistent with this notion, previous studies indicated that dynamic facial expressions, as compared with static ones, had facilitative effects on facial mimicry [20]–[22], emotion elicitation [23], and emotion recognition [24], [25]. Functional neuroimaging studies also showed that dynamic, compared with static, facial expressions enhanced the activity of the several brain regions (e.g., [26], [27]) as well as the functional connectivity between the brain regions [28], [29]. Based on these data, we hypothesized that the dynamic presentations of facial expressions would more clearly reveal the relationships among facial mimicry, emotion elicitation, and emotion recognition than would the static presentations.

To test these hypotheses, we re-analyzed the facial EMG and ratings data obtained in two studies (Study 1: [30]; Study 2: [22]). In both studies, facial EMG data were recorded from the corrugator supercilii and zygomatic major muscles while participants passively viewed facial expressions depicting negative and positive emotions presented dynamically and statically. Psychological ratings in response to stimulus facial expressions were also measured using the valence and arousal scales. We measured the experienced emotion in Study 1 and the experienced and recognized emotions in Study 2. We first checked bivariate relationships by caluculating correlations. Then, we conducted path analyses. We tested the hypothesis that the congruent facial muscle activity would elicit emotional experience and that the experienced emotion would induce emotion recognition. We predicted that the model would be supported when the ratings relied on the valence scales and the stimuli were presented dynamically.

## Methods

### Ethics Statement

This study was approved by the local ethics committee of Primate Research Institute, Kyoto University.

### Participants

Data were collected from 38 subjects in Study 1 (33 females and five males; mean age ± *SD*, 20.7±0.8 years) and 29 subjects in Study 2 (18 females and 11 males; mean age ± *SD*, 20.9±0.9 years), all of whom were Japanese volunteers.

### Stimuli

The stimuli in both studies consisted of video clips of amateur Japanese models. Stimuli depicting multiple models were prepared (four for Study 1 and eight for Study 2) as in previous studies (e.g., [12]). None of the faces was familiar to any of the participants. The models portrayed negative facial expressions (i.e., anger and sadness in Study 1 and anger in Study 2) and positive facial expressions (i.e., excitement and relaxation in Study 1 and happiness in Study 2). Under the dynamic presentation condition, participants were exposed to videos of facial expressions depicting changes from neutral to peak emotional expressions (mean presentation durations: 920 ms in Study 1 and 1,520 ms in Study 2). Under the static presentation condition, the final expressions under the dynamic expression condition were shown for the same duration of time as under the dynamic condition. The stimuli subtended a visual angle of about 16.5° in height ×11.0° in width.

### Procedure

The procedures used in Studies 1 and 2 were almost identical. EMG recordings were performed while participants passively viewed the stimuli. In total, 32 trials were conducted. EMG measures were taken for the corrugator supercilii and zygomatic major muscles using Ag/AgCl electrodes. A ground electrode was placed on the forehead. Data were amplified and filtered by a polygraph (Synafit 1000, NEC).

After EMG recordings, the stimuli were again presented to the participants, and they rated each stimulus using the affect grid [31], which graphically assessed valence from 1 (negative) to 9 (positive) and arousal from 1 (sleepiness) to 9 (high arousal). Study 1 included ratings for experienced emotion (i.e., the strength of the emotion felt by the participant upon perceiving the model's expression). Study 2 involved ratings for both experienced emotion and recognized emotion (i.e., the strength of the emotion that the participant recognized in the model's expression).

### Data analysis

In both studies, EMG data were analyzed using Psychophysiological Analysis Software 3.3 (Computational Neuroscience Laboratory of the Salk Institute). The data were sampled for 3,500 ms in each trial, which consisted of pre-stimulus baseline data obtained during 1,000 ms (the fixation point was presented) and data collected during the 2,500 ms after stimulus onset. After eliminating artifacts, differences in the mean absolute amplitudes of pre- and post-stimulus periods were calculated as the EMG data. The EMG data were then averaged under each condition for each participant. The EMG data for the corrugator supercilii and zygomatic major, experienced valence and arousal, and recognized valence and arousal were standardized for the dynamic or static presentation condition in each study.

To test the relationships between these variables, correlations were calculated for all pairs. Because several previous studies have reported quadratic as well as linear relationships between experienced valence and zygomatic major activity [14]–[16], we tested both linear and quadratic relationships. To check bivariate relationships, we also constructed scatterplots.

**Figure 1:**
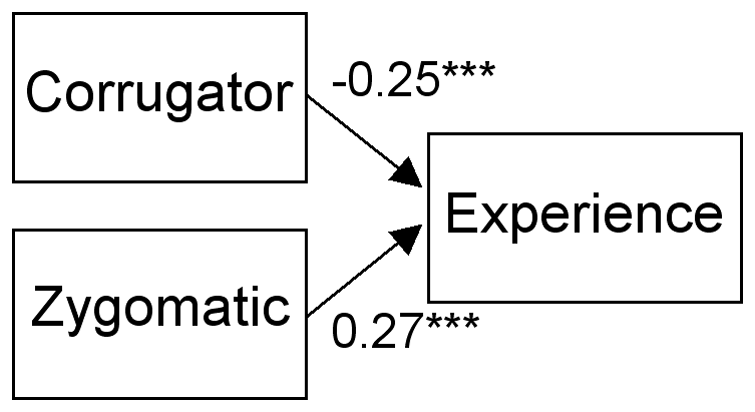
Standardized path coefficients for the valence ratings under the dynamic condition in Study 1. *** *p*<0.001.

**Figure 2:**
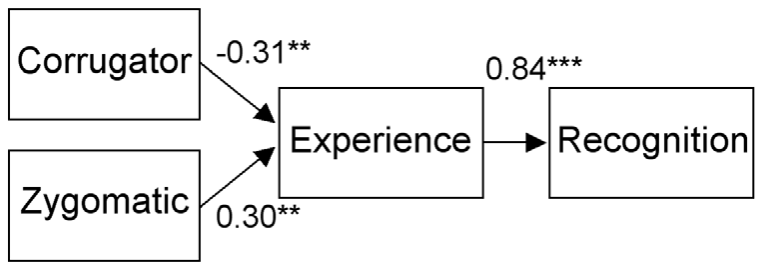
Standardized path coefficients for the valence ratings under the dynamic condition in Study 2. *** *p*<0.001; ** *p*<0.01.

Next, path analyses were conducted for the EMG and rating data using structural equation modeling (SEM) with generalized least square (GLS) estimation. Compared with the widely used maximum-likelihood estimation for SEM, GLS can be powerful for analyzing data from small samples [32], [33]. The SEM was implemented using Amos 16.0 (SmallWaters). Data obtained under the dynamic and static conditions were analyzed separately. Based on the results of correlations, we analyzed only linear relationships. In Study 1, data on valence and arousal were analyzed separately. Tested paths were from the corrugator supercilii to experienced emotion and from the zygomatic major to experienced emotion (Fig. 1). Analyses in Study 2 were also conducted separately for valence and arousal ratings. Tested paths were from the corrugator supercilii to experienced emotion, from the zygomatic major to experienced emotion, and from experienced emotion to recognized emotion (Fig. 2). We also compared the full model with partial models. For the analysis of the combined data from Studies 1 and 2, the valence and arousal ratings were simultaneously analyzed, and tested paths were from the corrugator supercilii and the zygomatic major to experienced valence and arousal. Path coefficients were tested for differences from zero using Wald tests. Path coefficients were also compared between dynamic and static presentation conditions. Based on our specific predictions, the analyses of the valence ratings under the dynamic presentation condition were performed using one-tailed statistics. Analyses of the arousal and the static presentation conditions were examined using two-tailed statistics. To evaluate the fit of the model, we computed χ^2^, the adjusted goodness-of-fit index (AGFI), and the root mean square error of approximation (RMSEA). The models were regarded well fitted with non-singnificant χ^2^, AGFI >0.90, and RMSEA <0.05 [34]. To compare models, the Akaike information criterion (AIC) was computed.

## Results

### Correlation

Correlation analyses of data obtained in Study 1 (Table 1; Fig. S1) and in Study 2 (Table 2; Fig. S2) showed that, under the dynamic presentation condition, all predicted relationships were significant (*p*<0.05). Specifically, higher corrugator supercilii activity was related with lower (more negative) experienced and recognized valence; higher zygomatic major activity was associated with higher experienced and recognized valence; and experienced and recognized valence were positively correlated. Similar but less evident correlations were found under the static presentation condition. Quadratic relationships between zygomatic major activity and experienced/recognized valence were not significant, except for a marginally significant correlation between squared zygomatic major activity and experienced valence under the dynamic condition in Study 1 (*p*<0.1).

**Table 1:** Correlation coefficients in Study 1.

		1		2		3		4		5		6	7	
Dynamic														
1	Experienced Valence													
2	Experienced Arousal	0.09												
3	Corrugator	−0.26	**	−0.07										
4	Zygomatic	0.28	***	0.07		−0.05								
5	(Experienced Valence)^2^	0.04		0.50	***	−0.12		0.07						
6	(Experienced Arousal)^2^	0.16	*	−0.06		0.06		0.23	**	0.12				
7	(Corrugator)^2^	0.02		0.02		−0.20	*	−0.13		0.14	+	−0.02		
8	(Zygomatic)^2^	0.14	+	−0.03		−0.06		0.71	***	−0.21		0.11	−0.19	
Static														
1	Experienced Valence													
2	Experienced Arousal	0.06												
3	Corrugator	−0.17	*	−0.11										
4	Zygomatic	0.18	*	0.01		−0.19	*							
5	(Experienced Valence)^2^	−0.04		0.47	***	−0.15	+	0.08						
6	(Experienced Arousal)^2^	0.03		−0.06		0.06		0.03		0.17	*			
7	(Corrugator)^2^	0.00		−0.09		−0.27	***	0.12		0.01		−0.02		
8	(Zygomatic)^2^	0.10		0.04		−0.12		0.32	***	−0.04		−0.11	0.30	***

All measures were standardized.

*** *p*<.001; ** *p*<.01; * *p*<.05; + *p*<.1.

**Table 2:** Correlation coefficients in Study 2.

		1		2		3		4		5		6		7		8	9	10		11
Dynamic																				
1	Experienced Valence																			
2	Experienced Arousal	−0.04																		
3	Recognized Valence	0.86	***	−0.08																
4	Recognized Arousal	0.07		0.28	*	0.09														
5	Corrugator	−0.33	*	0.05		−0.37	**	−0.18												
6	Zygomatic	0.33	*	0.19		0.32	*	0.31	*	−0.15										
7	(Experienced Valence)^2^	0.06		0.08		−0.02		0.13		−0.09		0.06								
8	(Experienced Arousal)^2^	−0.28	*	0.00		−0.26	+	0.15		−0.01		−0.05		0.16						
9	(Recognized Valence)^2^	−0.01		0.05		0.09		0.19		−0.01		−0.03		0.40	**	0.05				
10	(Recognized Arousal)^2^	0.08		0.22		0.04		−0.15		0.24	+	0.27	*	0.01		−0.11	−0.05			
11	(Corrugator)^2^	−0.10		0.05		−0.11		−0.21		0.54	***	0.10		−0.17		−0.11	−0.11	0.40	**	
12	(Zygomatic)^2^	0.22		0.21		0.17		0.31	*	−0.06		0.81	***	0.14		−0.01	−0.09	0.24	+	0.00
Static																				
1	Experienced Valence																			
2	Experienced Arousal	0.32	*																	
3	Recognized Valence	0.88	***	0.28	*															
4	Recognized Arousal	0.26	+	0.13		0.32	*													
5	Corrugator	−0.12		−0.02		−0.17		−0.23	+											
6	Zygomatic	0.25	+	0.01		0.23	+	0.01		−0.08										
7	(Experienced Valence)^2^	0.01		0.17		−0.02		0.06		0.06		0.03								
8	(Experienced Arousal)^2^	−0.04		−0.20		−0.08		0.06		0.03		0.03		0.17						
9	(Recognized Valence)^2^	−0.08		0.05		−0.09		0.11		0.02		0.09		0.52	***	0.03				
10	(Recognized Arousal)^2^	−0.04		0.09		0.02		0.08		0.07		0.23	+	0.11		0.12	0.12			
11	(Corrugator)^2^	−0.08		0.00		−0.03		−0.05		0.03		0.02		−0.16		−0.14	−0.14	0.05		
12	(Zygomatic)^2^	0.14		0.22		0.16		0.10		−0.01		0.67	***	0.06		0.10	0.10	0.15		−0.06

All measures were standardized.

*** *p*<.001; ** *p*<.01; * *p*<.05; + *p*<.1.

### Path analysis

Path analyses were conducted for dynamic and static presentation conditions separately. Because the correlation analyses did not reveal quadratic relationships between variables, we limited our analyses to linear relationships.

In Study 1, the analyzed model assumed that the congruent facial muscle activity elicited emotional experience (Fig. 1). Separate analyses were conducted for valence and arousal. For the analysis of valence under the dynamic condition, the Wald tests of path coefficients showed that both paths, from the corrugator supercilii to the experienced valence (negative) and from the zygomatic major to the experienced valence (positive), were significant (*p*<0.001; Table 3). The fit indices for the model were also highly acceptable. With the exception of the static presentation condition, under which the path from the corrugator supercilii to the valence was marginally significant (*p*<0.1), we found no significant paths involving the other conditions (*p*>0.1). When the path coefficients under the dynamic and static presentation conditions were compared, no path was significant (*p*>0.1).

**Table 3:** Path coefficients (with *SE*) and fit indices in Study 1.

Rating	Presentation	Path Coefficients							Fit Indices				
			Corrugator			Zygomatic							
			→ Experience			→ Experience			χ^2^		AGFI	RMSEA	
Valence	Dynamic		−0.25	(0.08)	***	0.27	(0.08)	***	0.31		0.99	0.00	
	Static		−0.15	(0.09)	+	0.14	(0.09)		5.14	*	0.86	0.17	
Arousal	Dynamic		−0.07	(0.08)		0.06	(0.08)		0.31		0.99	0.00	
	Static		−0.11	(0.09)		−0.01	(0.09)		5.14	*	0.86	0.17	

*** *p*<0.001; * *p*<0.05; + *p*<0.1.

In Study 2, the analyzed model assumed that the congruent facial muscle activity elicited emotional experience and that experienced emotion then induced emotion recognition (Fig. 2). Valence and arousal ratings were analyzed separately. As in Study 1, the analysis of the valence ratings under the dynamic condition showed all paths to be significant (*p*<0.001), and the fit of the model was sufficient (Table 4). There were no other conditions showing that all paths were significant, although the paths from experienced valence to recognized valence under the static condition and from experienced arousal to recognized arousal under the dynamic condition were significant (*p*<0.05) and the path from the zygomatic major to experienced valence under the static condition was marginally significant (*p*<0.1). Comparisons of the path coefficients under the dynamic and static conditions did not reveal any significant differences (*p*>0.1).

**Table 4:** Path coefficients (with *SE*) and fit indices in Study 2.

Rating	Presentation	Path Coefficients									Fit Indices		
		Corrugator			Zygomatic			Experience					
		→ Experience			→ Experience			→ Recognition			χ^2^	AGFI	RMSEA
Valence	Dynamic	−0.31	(0.13)	**	0.30	(0.13)	**	0.84	(0.07)	***	3.09	0.91	0.02
	Static	−0.11	(0.14)		0.25	(0.13)	+	0.87	(0.06)	***	1.36	0.96	0.00
Arousal	Dynamic	0.06	(0.14)		0.21	(0.15)		0.28	(0.13)	*	5.35	0.84	0.12
	Static	−0.02	(0.15)		0.00	(0.14)		0.13	(0.13)		3.13	0.91	0.03

*** *p*<0.001; ** *p*<0.01; * *p*<0.05; *p*+<0.1.

With respect to the model used in Study 2, which reflected our entire hypothesis, we tested the contribution of all paths to our findings. We compared the models with full versus partial paths. Based on the above full−model analysis, we analyzed the valence ratings under the dynamic presentation condition. The model comparisons using AIC indicated that the full model was optimal to account for the data (Table 5). All partial models showed higher AIC values than did the full model (i.e., less fit) and additional undesirable fit indices.

**Table 5:** Fit indices in Study 2.

Model	χ^2^		AGFI	RMSEA	AIC
C→E, Z→E, E→R	3.09		0.91	0.02	17.09
C→E, Z→E	24.28	***	0.47	0.30	36.28
C→E, E→R	7.73	+	0.83	0.13	19.73
Z→E, E→R	7.22		0.84	0.12	19.22
C→E	24.31	***	0.57	0.26	34.31
Z→E	24.62	***	0.57	0.26	34.62
E→R	10.64	+	0.81	0.14	20.64

C  =  Corrugator; Z  =  Zygomatic; E  =  Experience; R  =  Recognition.

*** *p*<0.001; + *p*<0.1.

To test the robustness of the model with a relatively large sample, we tested the model from Study 1 by combining the sample from Study 1 with that from Study 2. Because increasing the sample size allowed examination of more complex models, we simultaneously analyzed valence and arousal ratings. As in Study 1, analysis of data gathered under the dynamic condition showed significant paths from the corrugator supercilii and zygomaticus major to experienced valence (*p*<0.001), but not to experienced arousal (*p*>0.1) (Table 6). The fit indices of the model were high. With respect to data collected under the static condition, the path from the zygomatic major to experienced valence was significant (*p*<0.5), and that from the corrugator supercilii to experienced valence was marginally significant (*p*<0.1), although the fit indices were not acceptable. In terms of the comparison between the dynamic and static presentation conditions, the path from the corrugator supercilii to experienced valence was marginally significant (*p*<0.1).

**Table 6:** Path coefficients (with *SE*) and fit indices for the analyses of combined data.

Presentation	Path Coefficients												Fit Indices			
	Corrugator			Zygomatic			Corrugator			Zygomatic						
	→ Valence			→ Valence			→ Arousal			→Arousal			χ^2^		AGFI	RMSEA
Dynamic	−0.26	(0.07)	***	0.28	(0.07)	***	−0.03	(0.07)		0.10	(0.07)		1.16		0.99	0.00
Static	−0.13	(0.07)	+	0.18	(0.07)	*	−0.08	(0.07)		−0.01	(0.07)		8.12	*	0.90	0.12

*** *p*<0.001; * *p*<0.05; + *p*<0.1.

## Discussion

The analyses of data from Study 1, Study 2, and the additional dataset including data from both Study 1 and Study 2 showed that facial EMG activity predicted experienced emotional valence in response to dynamic facial expressions. Specifically, the activity of the corrugator supercilii and zygomatic major muscles were negatively and positively related to experienced emotional valences from negative to positive, respectively. The results of Study 2 further showed that experienced emotional valence predicted recognized emotional valence for dynamic facial expressions. These results support the theoretical model originally proposed by Nietzsche [3] and refined by several psychological researchers (e.g., [6]). The results are consistent with those of previous experiments that tested the partial paths within the model (e.g., [7]), although no empirical support for the full model had been reported until now. Our results also corroborate those of previous studies testing the relationships between facial EMG activity and experienced emotional valence using scenery stimuli (e.g., [14]). However, these studies did not specifically test the facial-expression stimuli and the relationships between experienced and recognized emotion. To our knowledege, this is the first study to demonstrate that facial mimicry influences the experience and recognition of emotional valence.

Our results are inconsistent with those of several previous studies that tested the relationships between these variables and reported null results [10]–[12]. The disparity in the results may be attributable to certain methodological differences. For example, as mentioned in the Introduction, although some studies [10], [12] used categorical accuracy scores to measure emotion recognition, we used ratings of emotional valence. Whereas none of these studies analyzed the EMG activity of the zygomatic major muscle, which has been implicated in studies uisng emotional scenes (e.g., [14]), we focused on the activty of this muscle. Although all the previous studies analyzed facial reaction data collected during more than 10 s, we analyzed data collected during less than 2.5 s and thus may have detected event-related reactions. Whereas all the previous studies conducted a series of correlation or regression analyses to test the relationships among variables, we performed SEM, which allowed more efficient and flexible comparisons between models (cf. [35]). We believe that our methods heightened the sensitivity to relationships among facial mimicry, experienced emotion, and recognized emotion.

Our results did not show relationships between facial EMG activity and experienced/recognized arousal. These results are consistent with those of previous studies investigating relationships between facial EMG activity and emotion experienced [14], [17]. The previous studies found that the scenes that elicited high levels of arousal induced strong autonomic activity, demonstrating positive correlations between these phenomena [14], [15]. In terms of autonomic activity, a previous study showed that the observation of emotional facial expressions elicited autonomic reactions corresponding to those depicted in the stimuli [36]. Based on these data, we speculate that, as in the case of facial mimicry, autonomic mimicry occurs in response to others' emotional facial expressions and then contributes to the experience and recognition of emotional arousal.

The results from correlational and path analyses revealed linear relationships between facial EMG activity and experienced valence ratings. These results are consistent with those of previous studies using scenery stimuli [14]–[16], [18]. However, although some of these studies also reported quadratic relationships between zygomatic major activity and experienced valence [14]–[16], our results did not show such patterns. This discrepancy may be attributable to differences in stimuli. A previous study suggested that greater zygomatic major activity in response to very positive scenes than to moderately positive scenes contributed to quadratic relationships between valence and zygomatic activity [16]. Because emotional facial expressions could elicit moderately valenced emotions compared with some scenes [14], our stimuli may have revealed only linear relationships.

The results of path analyses suggest that Nietzsche's [3] model is more appropriate for data obtained in response to dynamic than to static facial expressions. This is reasonable because his model would have been based on observations of natural social communications, which favor dynamic over static facial expressions. The results are also consistent with those of previous experimental studies showing that the dynamic presentations of facial expressions facilitated various types of psychological processes, including facial mimicry and the elicitation and recognition of emotions (e.g., [25]). Our results extend these findings, indicating that dynamic facial expressions have facilitative effects not only on several psychological processes but also on the links between these processes. However, it must be noted that our results also lent some support for the relationships between variables under the static condition, which were similar with those in the dynamic condition. We speculate that the psychological mechanisms involved in processing dynamic and static facial expressions may not differ qualitatively but that the former expressions may be more natural and therefore more powerful in their ability to elicit reactions.

The relationships observed between facial mimicry and psychological processes under the dynamic condition may also be consistent with evidence from neuroscientific studies. Previous functional neuroimaging studies have reported that the observation of dynamic facial expressions elicited greater activation in several brain regions, including the inferior frontal gyrus, than did the observation of static facial expressions. (e.g., [27]). Single-unit-recording studies in monkeys have shown that the monkey ventral premotor cortex, which has been proposed as a homologue of the human inferior frontal gyrus, contains specific neurons (“mirror neurons”) that discharge while individuals observe others' actions as well as when they execute actions themselves [37]. Consistent with such data, studies in humans have shown that the inferior frontal gyrus was active while participants imitated dynamic facial expressions [38], and electric stimulation around this region elicited automatic facial movements [39]. These data suggest that mirror neurons are activated specifically in the processing of dynamic facial expressions and that they match the observation with the execution of facial expressions, resulting in congruent facial muscle activity. In addition to inferior frontal gyrus activation, previous neuroimaging studies (e.g., [27]) have reported greater activation in response to dynamic than to static facial expressions in the amygdala, which was reported to be involved in the elicitation of emotions [40], and in the superior temporal sulcus, which was implicated in inferring others' emotional states [41]. Furthermore, several recent studies have reported that observation of dynamic, compared with static, facial expressions enhanced the functional couplings between these regions [28], [29]. Taken together, these data suggest the possibility that heightened activity and connectivity of the brain regions related to mirror neurons may underlie the enhanced relationships among facial mimicry, emotional experience, and emotion recognition involved in processing dynamic facial expressions. However, it should be noted that current information about the neural mechanisms of this phenomenon is insufficient. Some researchers have reviewed the literature and proposed hypotheses about the role of the mirror neurons in the neural mechanisms involved in emotional understanding (e.g., [42], [43]), but the details of these hypotheses appear to be inconsistent. Future research should investigate the neural substrates of the psychological phenomena observed in the present study.

One limitation of the present study should be acknowledged. Although our SEM analysis revealed association between variables and provided support for the model including causal assumptions, it did not fulfill the necessary conditions for causal inference [44]. For example, it is possible that the activity of facial muscles may not be the cause but the result of the elicitation of emotions. That is, participants may have displayed emotional expressions in response to elicited emotions via cognitive appraisals of others' emotions (cf. [45]). It is possible to test the causal directions of reciprocal relationships using SEM, but only when specific conditions are met, such as the use of instrumental variables (i.e., variables that are related to the cause but not to the effect) [46], [47]. Other criteria for the establishment of causality have also been proposed including temporal precedence (showing that a cause precedes an effect) and ruling out all extraneous variables [44], [48]. In terms of temporal precedence, data on the temporal sequence of relevant variables may be obtained by simultaneously recording facial EMG and continuously rating emotional and recognition experiences. With respect to extraneous variables, researchers should investigate as many candidates as possible. It must be noted that no statistical procedure can logically ensure the conditions necessary for causal inference [44]. However, it would be valuable to increase the empirical evidence related to causal relationships among facial muscle activity, elicitation of emotions, and recognition of emotions. We believe that our data represent a significant step in this direction.

In summary, the results of path analyses revealed that the congruent facial EMG activity predicted the experienced valence ratings, and the experienced valence then predicted the recognized valence in response to dynamic facial expressions. These results suggest that facial mimicry influences the sharing and recognition of emotional valence in response to others' dynamic facial expressions.

## Supporting Information

Figure S1 **Relationships among variables in Study 1. Scatter plots and regression lines are shown.**
(TIF)

Figure S2 **Relationships among variables in Study 2. Scatter plots and regression lines are shown.**
(TIF)
