# Supplementary figures and images for: Relationships among Facial Mimicry, Emotional Experience, and Emotion Recognition
Source: PLoS One. 2013 Mar 25;8(3):e57889. doi: 10.1371/journal.pone.0057889 (PMC3607589; doi:10.1371/journal.pone.0057889)

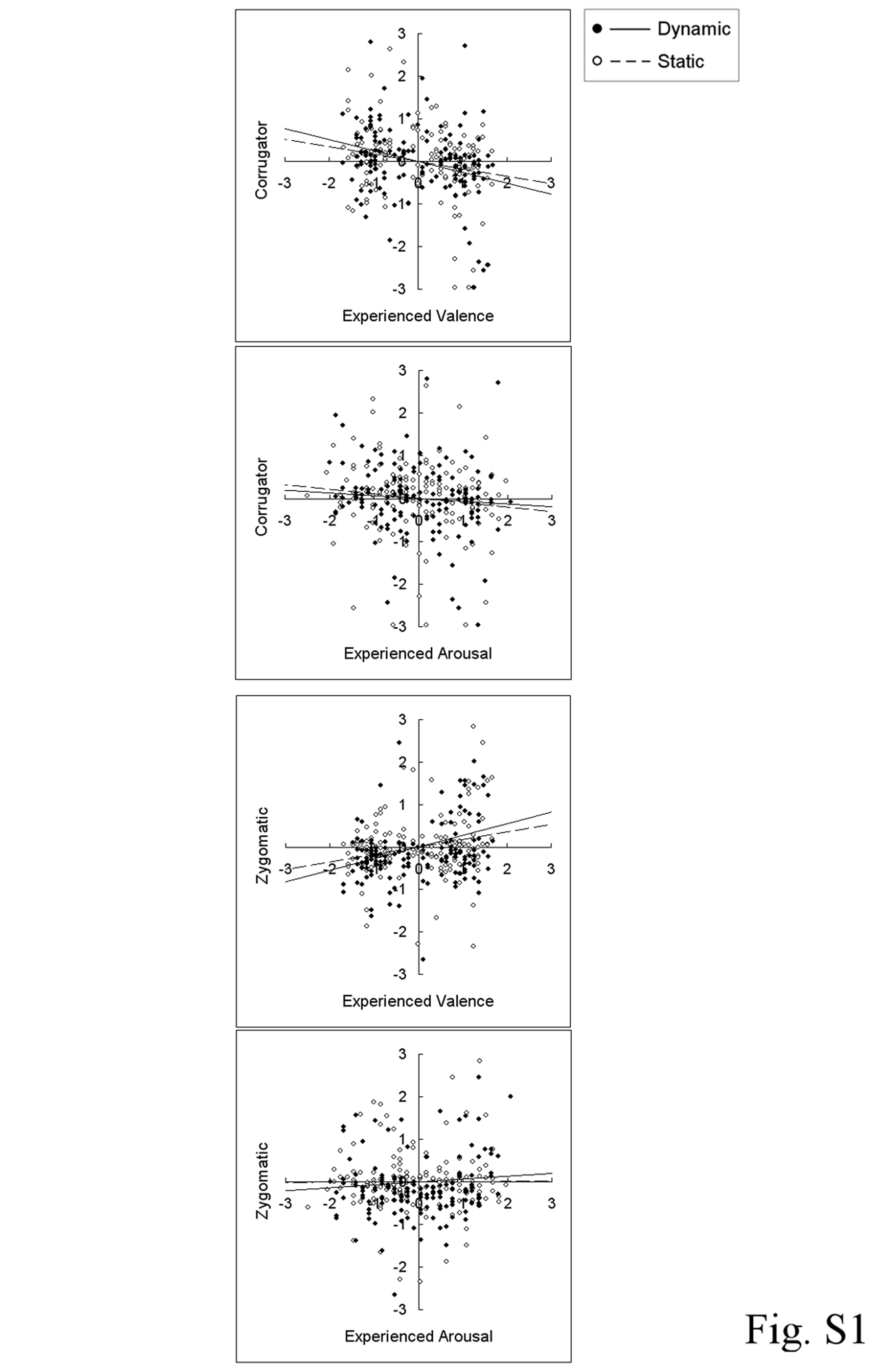

Supplement: Figure S1 — Relationships among variables in Study 1. Scatter plots and regression lines are shown. (TIF) [file pone.0057889.s001.tif]

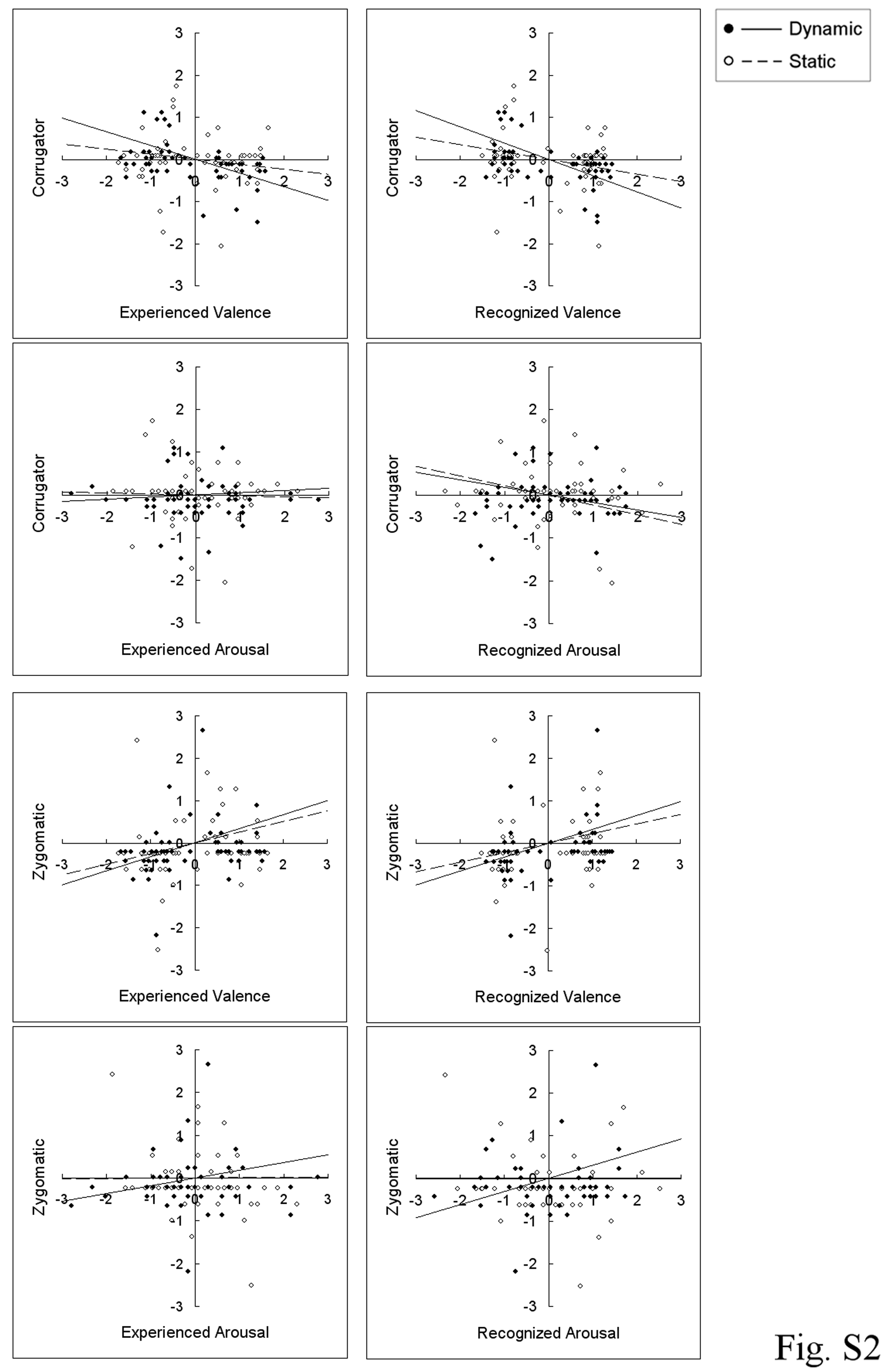

Supplement: Figure S2 — Relationships among variables in Study 2. Scatter plots and regression lines are shown. (TIF) [file pone.0057889.s002.tif]
